# Emergency Department and Overcrowding During COVID-19 Outbreak; a Letter to Editor

**DOI:** 10.22037/aaem.v9i1.1167

**Published:** 2021-03-25

**Authors:** Jean-Baptiste Bouillon-Minois, Julien Raconnat, Maelys Clinchamps, Jeannot Schmidt, Frédéric Dutheil

**Affiliations:** 1 Emergency Medicine, CHU Clermont-Ferrand, Université Clermont Auvergne, F–63000 Clermont–Ferrand, France.; 2 CNRS, LaPSCo, Physiological and Psychosocial Stress, F–63000 Clermont–Ferrand, France.; 3 Preventive and Occupational Medicine, CHU Clermont-Ferrand, F-63000 Clermont-Ferrand, France.

**Dear editor;**

Emergency Physicians (EPs) work under extreme stress conditions (1). Overcrowding – defined as hospital beds not being available for several patients who need one – has been a significant public health problem for more than a decade and is the consequence of the increase in health care demand and the decrease in bed spaces and number of staff (2). These parameters increase stress at work, which leads EPs to experience significant stress consequences, a feeling of diminished skills, and loss of time control (3). Many studies are interested in the overcrowding problem, like a recent study by Tangkulpanich et al. who found the predictive factors of revisiting in 48 hours (4).

Since the end of 2019, because of COVID-19, people were required to stay at home to prevent the spread of COVID-19 and the overflow of emergency and intensive care units. But in some places, there was no overflow, and a massive decrease, up to 50%, was observed in admission rate (5). In the rural area of Puy-de-Dôme – 653 742 inhabitants with a density of 82 inhabitants/km^2^ – only 335 patients had a positive test, with 30 deaths by July 1^st^, 2020. In the emergency department of Clermont-Ferrand – the principal city of Puy-de-Dôme –, there were 57 177 admissions in 2019 – i.e., a mean of 157 per day. Of those 57 177 emergency department admissions, 38 809 were discharged, and 18 368 (i.e., 50 per day) were hospitalized. But even if the hospital provided 21 851 available beds annually (60 per day), EPs spent 152 days on overcrowding.

Contrary to 2019, the year 2020 offered a massive decrease in the admission rate, with a maximum of 204 beds available on April 3^rd, ^2020. During the French COVID-19 lockdown between March 17^th^ and May 11^th^, there were no overcrowded days (55 days). As soon as the global lockdown ended, the emergency department was overcrowded again (Figure 1). Moreover, despite the cooling period of lockdown with a large number of beds available and few patients needing to be admitted, the year 2020 seemed harder than 2019. Indeed, 75 days out of the initial 185 days of the year (40.5%) were overcrowded in 2020, similar to 2019 with 79/185 overcrowding days (43%) during the first half of the year (Table 1). If we remove the 55 days of the COVID-19 lockdown, the percentage of overcrowded days rise up to about 58% in 2020. Although in-hospital mortality and hospital length of stay are correlated with the size of emergency department boarding (6), the number of hospitalization beds in France decreased from 468 000 in 2003 to 408 in 2015. 

In the same period, the number of admissions in the emergency departments increased from 15.5 million to 21 million per year in France.

Furthermore, emergency departments are identified as high-risk settings for medical errors and adverse events (7), concerning up to 10% of admissions. At the same time, several complaints are growing by approximately 1% per year, which may involve a rise of complementary investigations by fear of missing a diagnosis, increasing the time spent in emergency departments, and the stress of both patients and physicians (8). Interestingly, during the French COVID-19 lockdown, one author reported 32% decrease in transitory ischemic attacks, 64% in unstable angina, 42% in appendicitis, and 36% in seizures. However, we demonstrated that although the number of admissions hugely decreased (nearly by half) during the lockdown, the mean number of patients needing hospitalization during the first half of the year stayed similar (around 50 per day). Lastly, although the lockdown was not so problematic for emergency departments, the following months were a challenging period. During the French lockdown, planned surgery and medical consultations were canceled as much as possible and every surgeon, every staff member – emergency physician or not – was prepared to fight against COVID. But afterwards they wanted to reschedule the canceled surgeries and canceled meetings, and there were not more beds available for patients from emergency departments in hospitals. The months after the lockdown were tricky as the number of patients to hospitalize started to rise again, without downstream beds. 

**Table 1:** Number of hospitalizations needed and beds available per day and overcrowded days during first six months of the years 2019 and 2020

**Variables**	**2019 (185 days)**	**2020 (185 days)**	**p value**
**Hospitalizations/day**	51.0 ± 10.8	47.0 ± 10.1	<0.001
**Beds available/day**	59.0 ± 27.1	69.0 ± 46.6	<0.01
**Overcrowded (days)**	79 (42.7)	75 (40.5)	0.32

Data are presented as mean ± standard deviation of number (%).

**Figure 1:**
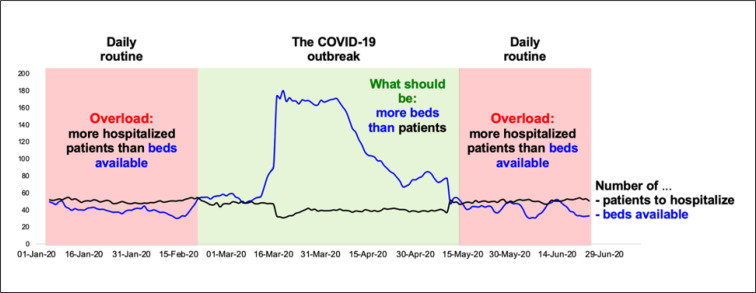
Overcrowding in the Emergency Department of Clermont-Ferrand between January 1^st^ and June 30^th^ 2020

## Conclusion:

Contrary to the common opinion, the COVID-19 lockdown was not the most terrible period for EPs. It even appeared that the lockdown could be considered the gold standard for patient care in emergency departments, without any problem to find a bed for those needing to be hospitalized. 

## Conflict of interest

The authors of this work declare no conflict of interest.

## Funding and support

Not applicable.
